# Barriers towards mental health service utilization among religious immigrants

**DOI:** 10.1192/j.eurpsy.2025.403

**Published:** 2025-08-26

**Authors:** R. Whitley, G. E. Jarvis

**Affiliations:** 1 Psychiatry, McGill University, Montreal, Canada

## Abstract

**Introduction:**

Evidence suggests that immigrants and minorities with mental distress underuse mental health services compared with the majority population. Such under-utilization, combined with other socio-cultural factors, can impede recovery from mental illness. The population of Canada is becoming increasingly diverse, primarily due to record levels of immigration. These immigrants come from a variety of traditions including Muslim, Christian, and Jewish backgrounds. For such immigrants, their religious beliefs, practices and activities are often central to their lives and influence important decisions regarding health and well-being. These changing demographics have prompted calls for more research and action regarding the mental health service experience of religious immigrants.

**Objectives:**

The objective of this study was to identify and understand self-identified barriers to mental health service utilization among religious immigrants, including eliciting experiences within the mental health care system.

**Methods:**

To meet these aims, we employed a qualitative community-based approach, conducting in-depth semi-structured interviews with 58 first- or second-generation immigrants to Canada who identified as people of religious faith, comprising Christians, Muslims and Jews. All participants had used a mental health service in recent years, and they reported a variety of mental disorders, mostly depression and anxiety. Interviews were transcribed and data was analyzed using thematic analysis techniques.

**Results:**

Analysis revealed three core barriers to service utilizations. First, participants often reported that some people in their social circle (such as parents and clergy) held stigmatizing views of mental illness, including sceptical views about the reality of mental illness. This contributed to self-stigma, inhibited disclosure and delayed help-seeking. Second, participants stated that service providers typically had a very limited understanding of the cultural and religious context of their lives, and sometimes conveyed a dismissive or ignorant attitude towards their deeply-held religious beliefs and practices. This negatively affected service utilization and the development of a therapeutic alliance. Third, some participants noted that they (and other members of their communities) lacked knowledge about mental illness, available treatments, and effective therapies, meaning they were unaware of potential services and supports.

**Conclusions:**

The results suggest an urgent need for a multi-pronged approach to better engage religious minorities with mental distress. On the clinical side, there is a need for more religious and cultural competence training for Canadian clinicians. On the community side, culturally-appropriate anti-stigma and mental health literacy interventions may need to be co-created and implemented in partnership with different immigrant communities in Canada.

**Disclosure of Interest:**

None Declared

